# The Evaluation and Validation of Blood-Derived Novel Biomarkers for Precise and Rapid Diagnosis of Tuberculosis in Areas With High-TB Burden

**DOI:** 10.3389/fmicb.2021.650567

**Published:** 2021-06-14

**Authors:** Zhen Gong, Yinzhong Gu, Kunlong Xiong, Jinxia Niu, Ruijuan Zheng, Bo Su, Lin Fan, Jianping Xie

**Affiliations:** ^1^State Key Laboratory Breeding Base of Eco-Environment and Bio-Resource of the Three Gorges Area, Key Laboratory of Eco-Environments in Three Gorges Reservoir Region, Ministry of Education, School of Life Sciences, Institute of Modern Biopharmaceuticals, Southwest University, Chongqing, China; ^2^Shanghai Key Laboratory of Tuberculosis, Shanghai Clinic and Research Center of Tuberculosis, Shanghai Pulmonary Hospital, Tongji University School of Medicine, Shanghai, China; ^3^College of Fisheries and Life Sciences, Shanghai Ocean University, Shanghai, China

**Keywords:** tuberculosis, blood biomarkers, BATF2, UBE2L6, VAMP5, SERPING1

## Abstract

Tuberculosis (TB) remains a highly contagious public health threat. Precise and prompt diagnosis and monitoring of treatment responses are urgently needed for clinics. To pursue novel and satisfied host blood-derived biomarkers, we streamlined a bioinformatic pipeline by integrating differentially expressed genes, a gene co-expression network, and short time-series analysis to mine the published transcriptomes derived from whole blood of TB patients in the GEO database, followed by validating the diagnostic performance of biomarkers in both independent datasets and blood samples of Chinese patients using quantitative real-time PCR (qRT-PCR). We found that four genes, namely UBE2L6 (Ubiquitin/ISG15-conjugating enzyme E2 L6), BATF2 (Basic leucine zipper transcriptional factor ATF-like), SERPING1 (Plasma protease C1 inhibitor), and VAMP5 (Vesicle-associated membrane protein 5), had high diagnostic value for active TB. The transcription levels of these four gene combinations can reach up to 88% sensitivity and 78% specificity (average) for the diagnosis of active TB; the highest sensitivity can achieve 100% by parallel of BATF2 and VAMP5, and the highest specificity can reach 89.5% through a combination of SERPIG1, UBE2L6, and VAMP5, which were significantly higher than 75.3% sensitivity and 69.1% specificity by T-SPOT.TB in the same patients. Quite unexpectedly, the gene set can assess the efficacy of anti-TB response and differentiate active TB from Latent TB infection. The data demonstrated these four biomarkers might have great potency and advantage over IGRAs in the diagnosis of TB.

## Introduction

Despite decades of vaccine immunization and anti-TB chemotherapy, tuberculosis (TB) caused by *Mycobacterium tuberculosis* (MTB) remains a devastating disease and an enormous burden to global public health, with around one fourth of the population at risk of being infected, about 10 million new TB incidences, and 1.2 million deaths worldwide in 2019 (WHO, 2019). Rapid and precise diagnosis of active TB largely represents an unmet clinical need (Pai and Schito, 2015). Traditional diagnosis has defects, such as the low sensitivity (12–15%) of acid-fast bacilli (AFB) and time-consuming nature of cultures (Akkaya and Kurtoglu, 2019). Molecular diagnostics such as Xpert MTB/RIF (Cepheid, Sunnyvale, CA, United States) can achieve a sensitivity of 34–66.7% for smear negative- Pulmonary TB (PTB) and extrapulmonary TB (Qureshi et al., 2019; Wu et al., 2019). Xpert MTB/RIF ultra can improve the sensitivity for TB but has decreased specificity compared with Xpert MTB/RIF (Wu et al., 2019; Jiang et al., 2020). Current etiological methods have limited sensitivity in smear-negative active TB, especially paucibacillary TB (Steingart et al., 2013). Therefore, etiological methods are not suitable for fast diagnosis of TB. Blood-derived biomarkers for precise and rapid diagnosis of TB are intensively studied to meet clinical needs.

The most applied blood-derived immunological method for diagnosis of TB was interferon-γ release assays (IGRAs) and tuberculin skin testing (TST), however, they cannot distinguish active TB from latent TB infection (LTBI) or HIV positive patients (Rangaka et al., 2012). Mycobacteria-specific cytokines are intensively explored as a biomarker to distinguish latent TB infection from active TB (Marc et al., 2015). The diagnostics based on biomarkers derived from blood samples have recently been intensively explored (Denkinger et al., 2015), and also meet WHO’s target product characteristics. The blood-based biomarkers’ diagnostics have great advantages (Sweeney and Khatri, 2016; Walter et al., 2016) for quick samples collection and quantification, as well as point-of-care tests (POCT) (Wallis et al., 2010). However, based on existing research results, effective biomarkers based on whole blood are still lacking.

**FIGURE 1:**
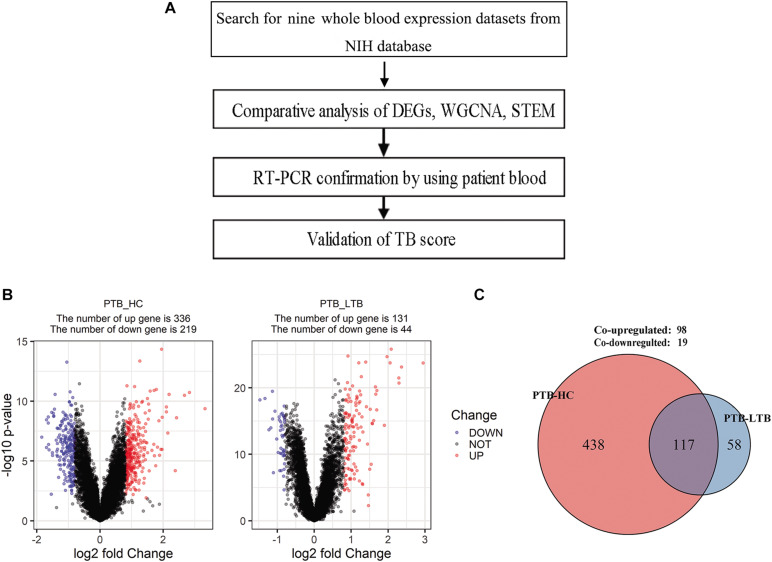
Schematic of analysis workflow and the differentially expressed genes in PTB compared with healthy control (HC) and LTB in GSE19491. **(A)** Schematic of analysis workflow, **(B)** Volcano plot of differentially expressed genes at adjusted *p* < 0.05 and |log2FC| > 0.8 for PTB-HC and PTB-LTB, **(C)** The number of differentially expressed genes shared by PTB-HC and PTB-LTB.

The host transcriptome response to MTB infection is a valuable source for this end, as exemplified by the abundant National Institutes of Health Gene Expression Omnibus (NIH GEO). To transform the transcriptome data into clinically actionable TB diagnostics, we curated nine transcriptome datasets based on the whole blood from NCBI that meet the statistical criteria for effective data analysis (Figure 1A). In combination with clinical sample analysis, we determined the specificity and sensitivity of the candidate in the diagnosis of active tuberculosis. The results showed that the effectiveness of the novel diagnostic biomarker was significantly better than T-SPOT.TB. In summary, in this study, a four-gene set (UBE2L6, BATF2, SERPING1, and VAMP5) was validated as a novel method for the diagnosis of active PTB, as well as a biomarker for monitoring anti-TB treatment efficacy.

## Materials and Methods

### Microarray Data Information and Usage in Discovery/Validation Stage

Gene Expression Omnibus (GEO) datasets from published microarray-based studies of PTB versus LTBI or other diseases were collected for data mining. From the nine datasets (GSE19491, GSE40553, GSE56153, GSE42834, GSE39941, GSE37250, GSE103119, GSE94438, and GSE124548), 2804 samples were obtained (Table 1). After screening for the most relevant and comprehensive blood samples, 1654 samples were kept for further study. However, most samples are highly heterologous for processing methods, and cannot be directly used for analysis. GSE19491 has very comprehensive information with a large number of samples and was assayed by the same laboratory with uniform methods. Multiple individuals were included in this dataset, such as PTB, LTBI, HC, and other pulmonary diseases. Additionally, the change of transcriptome during treatment monitoring was also analyzed. Specifically, three subseries (GSE19439, GSE19442, and GSE19444) in GSE19491 containing the transcriptome data of TB, LTB, and HC were used for gene differential expression and correlation analysis. Therefore, GSE19491 was used as the discovery dataset to find the differential expression by Limma, correlation by WGCNA, and time-course trend by STEM.

**TABLE 1:** Geo information used in this study.

	Year	Reference	Platform	Participants Age	Race distribution	Participants classification	Treatment
GSE19491 (SubSeries: GSE19435, GSE19439, GSE19442, GSE19444)	2010	Berry	GPL6947	Adults	African European	PTB, LTB, and HC;	3 time-points: 0, 2, 12 months in GSE19435
GSE40553	2012	Bloom	GPL10558	Adults	European	PTB	5 time-points: 0, 0.5, 2, 6, 12 months
GSE56153	2014	Ottenhoff	GPL6883	Adults	Asian	PTB, HC	3 time-points: Active, Treatment, Recover
GSE42834	2013	Bloom	GPL10558	Adults	–	PTB, lung cancer, other pulmonary diseases	–
GSE39941	2014	Anderson	GPL10558	Children	African	PTB, other diseases (HIV +/-)	–
GSE37250	2013	Anderson	GPL10558	Adults	African	PTB, other diseases (HIV +/-)	–
GSE103119	2018	Wallihan	GPL10558	Children	–	HC, pneumonia caused by bacterial or viral infections	–
GSE94438	2018	Thompson	GPL11154	Adults	African	household contact	2 time-points: 6, 18 months
GSE124548	2019	Kopp	GPL20301	Adults	–	pulmonary disease caused by cystic fibrosis	–

GSE40553 and GSE56153 contained the time-course transcriptome data of TB patients post treatment. GSE42834 contained patients with active TB or miscellaneous pulmonary diseases. GSE37250 and GSE39941 are samples from patients of TB and other diseases with or without HIV co-infection. GSE94438 samples are from household contact subjects (Suliman et al., 2018). Thence, we chose the six datasets for validation.

Another three datasets (GSE103119, GSE124548, and GSE42834) were used to validate the biomarker specificity. GSE103119 contained patients with pneumonia caused by bacteria or virus (except MTB) and healthy subjects, while GSE124548 samples are from cystic fibrosis patients, used to differentiate pulmonary diseases.

Datasets first underwent quantile normalization and were log2 transformed. We mapped the probes to gene symbols based on the probe data before Dec 5, 2018 from GEO.

### Identification of Biomarkers From Multiple Datasets

In order to discover the molecules most likely to be biomarkers of tuberculosis from the data set, we combined multiple data analysis methods: Differentially expressed genes (DEGs), Co-expression network analysis, and Time series analyses.

### Real-Time qPCR Validation of Differentially Expressed Genes by Prospective Clinical Study

Patients who met inclusive criteria were prospectively enrolled into this study from January 1, 2019 to July 31, 2019 in Shanghai Pulmonary Hospital. The study was approved by the Institutional Review Board of Shanghai Pulmonary Hospital, School of Medicine, Tongji University (approval number: K17-022) and the enrolled patients signed informed consent forms. Included patients donated 2 ml peripheral venous blood for RNA extraction.

The inclusion criteria are: patients diagnosed with pulmonary TB (PTB), lung cancer, or pneumonia; those who are serum HIV negative; and patients willing to be included in this study. Diagnostic standards were as followed: PTB was diagnosed by MTB MGIT 960 culture positive consistent with WHO guidelines of diagnosis and treatment on pulmonary tuberculosis (19); lung cancer was confirmed by pathology examination; and pneumonia was diagnosed according to the national guideline.

The exclusion criteria are: patients with an uncertain diagnosis, HIV positive patients, those taking immunosuppressive agents, cases complicated by cancer or other complications or other pulmonary diseases, or patients reluctant to attend the study.

Quantitative real-time PCR (qRT-PCR) was used to validate the differential expression of the four shortlisted genes in blood samples from participants. 2.0 mL peripheral venous blood was taken directly into PAXgene blood RNA tubes (PreAnalytiX, Hombrechtikon, Switzerland) and stored at −20°C for use. RNA was extracted from PAXgene tubes stored blood. Before analysis, all test samples and primers were assigned random numerical codes that masked the disease, control status, and the gene identity. The qRT-PCR-based validation and GEO data mining were done in a fully blind manner. The primers used are listed in Table 2. The gene expression levels were quantified relative to the transcription of β-actin by using an optimized comparative Ct (ΔΔCt) value method.

**TABLE 2:** Primers used in this study.

Genes	Primers
BATF2	F-CACCAGCAGCACGAGTCTC
(NM_138456.3)	R-TGTGCGAGGCAAACAGGAG
UBE2L6	F-CGCGCTGTGTCGCGG
(NM_004223.3)	R-GCAGGGGCTCCCTGATATTC
VAMP5	F-ATGCGTAACAACTTCGGCAAG
(NM_006634.2)	R-GGCCAGGTTCTGTGTAGTCTT
SERPING1	F-GGGATGCTTTGGTAGATTTCTCC
(NM_001032295.1)	R-GAGGATGCTCTCCAGGTTTGT
	R-ACAGTTGGTCCATAGCCTGC
β-actin	F-TTCCTTCCTGGGCATGGAGTCC
	R-TGGCGTACAGGTCTTTGCGG

### Validation of TB Score

By data mining and validating by clinical study, we defined the geometric mean of the four gene transcription levels as the TB score ($\text{TBscore }=\;\sqrt[{4}]{\text{UBE}2\text{L}6\text{*BATF}2\text{*SERPING}1\text{*VAMP}5})$ (Sweeney et al., 2016). This TB score was directly tested for diagnostic power by receiver operating characteristic (ROC) curves using the R package pROC. Violin plots showed the TB score for a dataset response to treatment at specific time points. Violin plot error bars showed the inter-quartile range (IQR) between non-normal distributions within subsets. Between-groups TB score comparisons were done with the Wilcoxon rank sum test. Significance levels were set at two-tailed *p* < 0.05. All computation and calculations were done in the R language (version 3.5.1).

### T-SPOT.TB Assay

T-SPOT.TB was performed in accordance with the manufacturer’s instructions (Oxford Immunotec Ltd.). Blood samples were collected immediately prior to the tests in order to avoid potential interferences, and patients who received blood transfusions or underwent positron emission tomography-computed tomography scans within 1 week of the test were recommended to undergo a second test 2 weeks later. Peripheral blood mononuclear cells (PBMCs) were separated from blood samples using Ficoll-Hypaque gradient centrifugation at 400 × *g* for 30 min at 20°C. PBMCs were seeded on precoated IFN-γ ELISpot plates and incubated with media without an antigen (as a negative control), media containing peptide antigens derived from ESAT-6 (labeled panel A) or peptide antigens derived from CFP-10 (labeled panel B), or media containing phytohemagglutinin (as a positive control) in a 5% CO2 atmosphere at 37°C for 20 h. 29–31. After counting the number of spot-forming cells, results are reported with negative control results subtracted (i.e., measured sfu number minus sfu number of negative control), according to the recommendations of the manufacturer. The values for ESAT-6 (panel A) and CFP-10 (panel B) were also scored individually using the same procedure and the maximum of them was regarded as the final result of T-SPOT.TB. All T-SPOT.TB testing was performed before the patients were prescribed anti-TB medications.

### Statistical Analysis

Statistical and machine learning methods (R packages: limma, WGCNA, pROC, and STEM software) were employed to discover and validate the biomarker genes for TB diagnosis and treatment response based on the mRNA levels in blood samples. The analyses were carried out using scripts written in Rstudio. The differences in gene expression levels between TB patients’ and healthy controls’ blood samples were compared using the Wilcoxon test. Multiple comparisons were carried out in patients with lung cancer, pneumonia, and TB by Kruskal-Wallis. Significance levels were set at *p* < 0.05.

## Results

### Four Candidate Biomarker Genes Were Found by Integrating the Results of DEGs, WGCNA, and STEM Analysis

The DEGs in the three subseries of GSE19491 were analyzed using the limma package following data preprocessing. A total of 555 DEGs were identified, including 336 up-regulated genes and 219 down-regulated genes in PTB compared to HC and 175 DEGs w including 131 up-regulated genes and 44 down-regulated genes in PTB compared to LTB (Figure 1B). Finally, 117 DEGs were found to be shared by both PTB-HC and PTB-LTB, containing 98 up-regulated genes and 19 down-regulated genes (Figure 1C).

**FIGURE 2:**
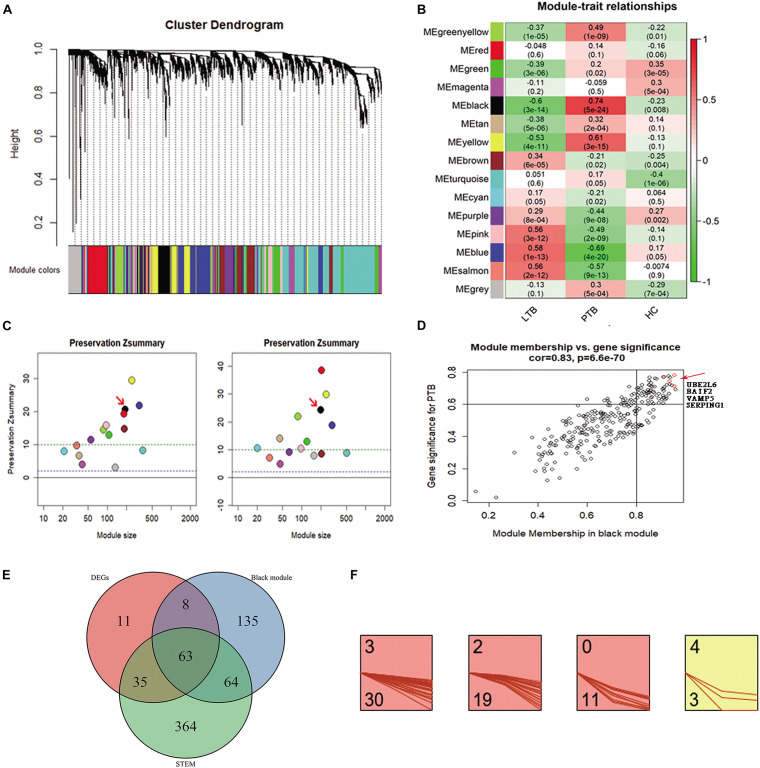
Co-expression analysis of WGCNA in the three subseries. **(A)** Cluster dendrogram showed that all genes were assigned to one of 14 modules. **(B)** Correlation between modules and traits. The upper number in each cell refers to the correlation coefficient of each module in the trait, and the lower number is the corresponding p-value. Among them, the black modules were the most relevant modules with TB traits. **(C)** The preservation Zsummary of the black module was 20 for GSE37250 (left), and 24 for GSE42834 (right), Zsummary > 10 indicates high preservation. **(D)** A scatter plot of GS for TB versus the MM in the Black module. Intramodular analysis of the genes found in the Black module, which contains genes highly correlated with PTB, with *p* = 6.6e-70 and correlation = 0.83. **(E)** There are 63 genes present in the DEGs, WGCNA black module, and STEM profiles. **(F)** 63 key genes, 30, 19, 11, and 3 were assigned to 3, 2, 0, and 4 profiles.

4807 genes in 134 samples were analyzed by WGCNA to find the modules of highly correlated genes. By a power of 8, 14 modules were found (Figure 2A). Among all modules, the black module with 270 genes had the highest correlation coefficient (*p* = 5e-24; *r* = 0.74) with PTB (Figure 2B). An intra-modular analysis of GS and MM of the genes in the black module found that GS and MM were significantly correlated (*p* = 6.6e-70; *r* = 0.83), further supporting that the genes in the black module were highly correlated (Figure 2D). To investigate whether these modules are conserved in our network, two independent datasets of GSE37250 and GSE42834 datasets were used to test the preservation of these modules. Zsummary > 10 indicates high preservation. Black modules in the GSE37250 and GSE42834 had Z-summary 20, 24, indicating they are well-preserved network in our study (Figure 2C).

To minimize the candidate genes for biomarkers, we conducted a comparative analysis and found 63 genes (Supplementary Table 1) present in the DEGs, WGCNA black module, and STEM profiles (Figure 2E). Of the 63 key genes, 30, 19, 11, and 3 were assigned to 3, 2, 0, and 4 profiles (Figure 2F). They were significantly enriched in immune response related terms in the GO, Reactome pathway, and Uniprot keywords enrichment analyses by STRING website (Szklarczyk et al., 2017). This result encouraged us to further explore the roles of the 63 genes in TB.

Too many genes might be counterproductive for rapid and precise biomarker diagnosis. We further shortlisted the 63 genes to four genes (SERPING1, BATF2, UBE2L6, and VAMP5), due to their highest GS and MM in the black module, reduced constantly in STEM profile 3, and differentially expressed in both the PTB versus LTB and PTB versus HC. Together, the transcriptional levels of the four genes correlated with TB and changed significantly during treatment. It implied that the four genes might play essential roles in the development of TB and could be candidates for new diagnostic biomarkers.

### Four Genes Showed Good Clinical Performance by Real-Time qPCR Validation in Peripheral Blood From Patients

To validate the clinical efficacy of the four genes, 150 participants were included; of them, 14 cases were excluded due to obscure diagnosis, and a total of 126 participants were finally enrolled into the study. They were classified into four groups: 51 cases with active PTB, 30 cases with pulmonary lung cancer (TUMOR), 30 cases with pneumonia (INFLA), and 15 cases as healthy donor (HC). Patient’s clinical characteristics were shown in Table 3.

**TABLE 3:** Patient’s information.

	TB	LC	Pulmonary inflammation	HP
Number of people	51	30	32	16
Gender (male, proportion)	78.4%	–	–	56.25%
Age (median, range, and years)	35 (20–83)	–	–	20–40
Date of inspection	1/2019 and 6/2019	7/2019	7/2019	7/2019

*TB, Tuberculosis; LC, Lung cancer; HP, Healthy population.*

The transcription levels of the four genes were detected by qRT-PCR. The results showed that BATF2, SERPIG1, UBE2L6, and VAMP5 were significantly increased in PTB compared with HC (Wilcoxon test, *P* < 0.05).

**FIGURE 3:**
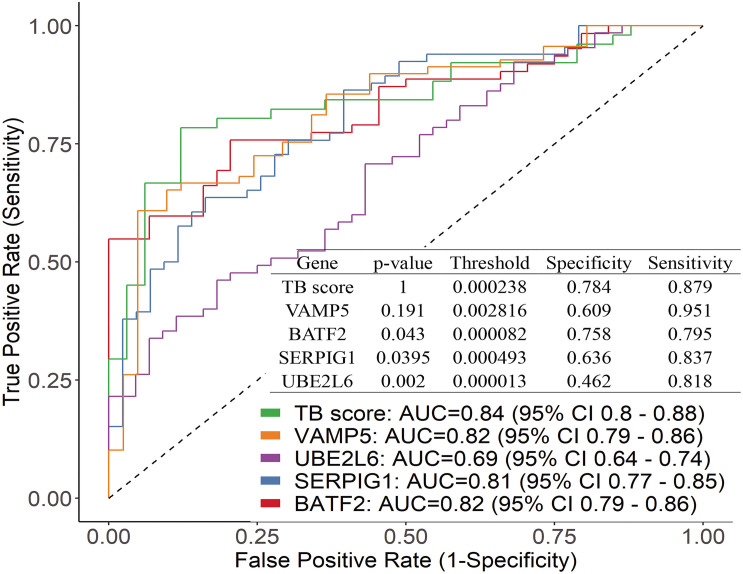
Performance of the genes by using ROC curve and the difference between the ROC curve of each gene and TB score.

We further plotted the ROC curve to evaluate diagnostic power (Figure 3). The results showed that the diagnostic power of a single gene was relatively lower than their combination and the ROC curve of each gene is different from these of TB score by genes combination (venkatraman method (Venkatraman, 2015), *P* < 0.05). In the patient samples, the combination of three genes (BATF2-SERPIG1-VAMP5 and BATF2-SERPIG1-UBE2L6) or two genes (BATF2-SERPIG1 and SERPIG1-VAMP5) can improve the diagnostic performance, and there was no difference in the ROC curve in between those matches with the four-gene combination (Supplementary Image 1). The results from Chinese patients were different from those reported in the GEO data in which the combination of four genes showed better performance. The performance of four gene combinations in Chinese patients can reach up to 100% for sensitivity or specificity, the average sensitivity or specificity is AUC = 0.84, sensitivity = 88%, and specificity = 78%, similar to that by pure GEO datasets analysis from a non-Chinese population, which has an AUC = 0.86, sensitivity = 86%, and specificity = 81%.

### The Diagnostic Efficacy of the Four Genes for Active TB Is Significantly Higher Than That of T-SPOT.TB Conducted Over the Same Patients

To compare the four candidate biomarkers and T.SPOT’s performance for the diagnosis of active TB, all patients were tested by T-SPOT.TB. 75.3% sensitivity and 69.1% specificity were found, suggesting that the diagnostic accuracy of T-SPOT.TB for active tuberculosis is significantly lower than the four genes in areas with high TB burden.

### Four Genes Have Good Specificity for Active TB Diagnosis

**FIGURE 4:**
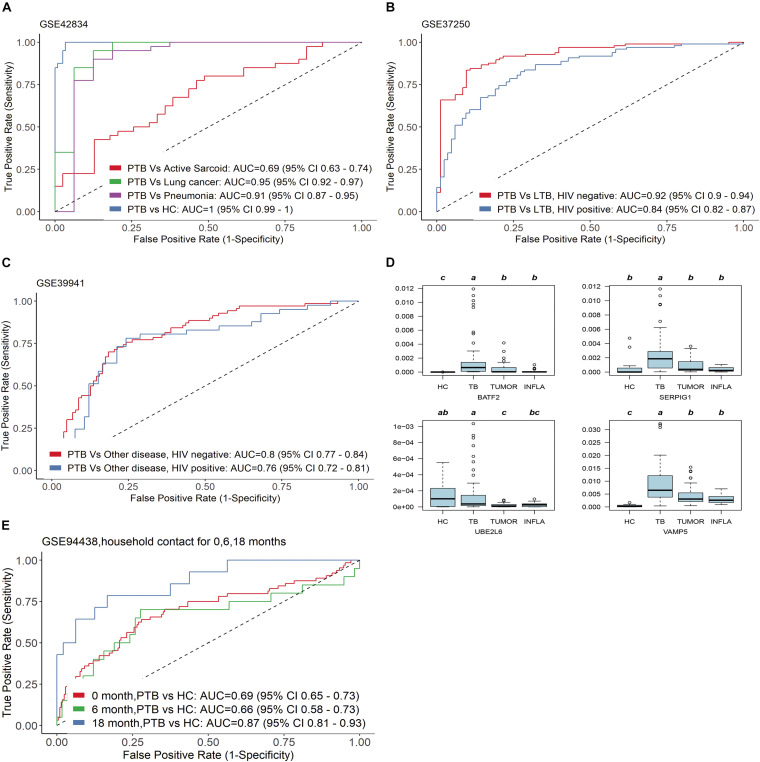
Four genes have good specificity for active TB diagnosis. **(A,D)** PTB versus HC and other pulmonary diseases, **(B)** active TB versus LTBI with HIV and active TB versus LTBI without HIV, **(C)** active TB versus other diseases with HIV or without HIV in GSE39941. **(E)** The four genes can predict whether close contacts of tuberculosis patients will develop active TB.

In the clinic, other lung diseases often confound the accuracy of tuberculosis diagnosis to a large extent. The diagnostic specificity of candidate markers is crucial. In order to verify the specificity of the four genes, we examined TB score in independent gene expression datasets from clinical TB samples, comparing its efficacy among four types of comparisons by ROC curve (Figure 4): (1) PTB versus HC [AUC 1.00 (95% CI 0.99–1.00)] and other pulmonary diseases : PTB versus sarcoidosis AUC 0.69 (95% CI 0.63–0.74); PTB versus Lung cancer AUC 0.95 (95% CI 0.92–0.97); PTB versus Pneumonia AUC 0.91 (95% CI 0.87–0.95) in GSE42834 (Figure 4A); (2) active TB versus LTBI with HIV (AUC 0.84 (95% CI 0.82–0.87)] and active TB versus LTBI without HIV [AUC 0.92 (95% CI 0.90–0.94)] in GSE37250 (Figure 4B); (3) active TB versus other diseases with HIV [AUC 0.76 (0.72 −0.81)] or without HIV [AUC 0.80 (0.77–0.84)] in GSE39941 (Figure 4C); and (4) active TB patients response to treatment at specific time points (GSE40553 and GSE56153). The TB score did well across all conditions (mean AUC 0.86, sensitivity 86%, and specificity 81%) except sarcoidosis, which might be due to the common disease-related signatures in TB and sarcoidosis (Maertzdorf et al., 2012).

We further examined the transcription level of the four genes in blood samples from patients with tumors (TUMOR) and pneumonia (INFLA). The results showed that the transcription of the four genes in PTB was significantly higher than that in HC, TUMOR, and INFLA (Figure 4D). The transcription of SERPIG1 were about 3–7 times in PTB compared with HC, TUMOR, and INFLA. Those in HC, TUMOR, and INFLA were almost identical, but they were about 3–7 times in PTB compared with HC, TUMOR, and INFLA. The transcription levels of BATF2, UBE2L6, and VAMP5 in TUMOR and INFLA were also similar and were slightly higher than those in HC. However, the transcription levels of the above three genes in PTB were about 3–8 times higher than those in TUMOR and 3–15 times higher than that in INFLA (Supplementary Table 2).

In addition, the four genes can also predict whether close contacts of tuberculosis patients will develop active TB. According to GSE94438, TB score can effectively identify those who developed TB 18 months after contact with active tuberculosis [AUC 0.87 (95% CI 0.81–0.93)] (Figure 4E).

### The Four Genes Can Also Be Biomarkers for Treatment Efficacy and Differential Diagnosis *via* Cross-Validation of TB Score in Independent Test Datasets

**FIGURE 5:**
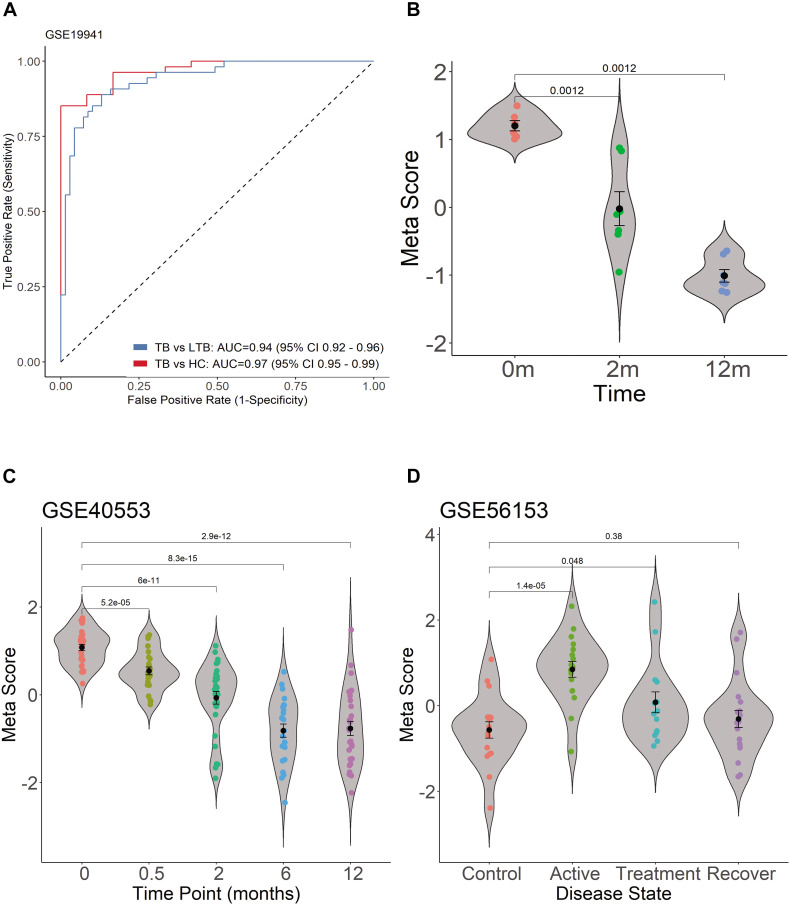
Diagnostic performance of TB score by using ROC curve in GSE19491 and violin plots in GSE19491, GSE40553, and GSE56153. **(A)** Four genes can distinguish PTB and HC, and can differentiate active PTB from LTBI. **(B)** The scores of TB patients decreased significantly after effective treatment. **(C)** The TB score was significantly decreased after treatment for PTB. **(D)** The TB scores of patients return to normal after treatment between healthy control and recovery patients.

As expected, when we investigated TB score in the discovery datasets, we found the four-gene set can differentiate active TB from HC with AUC 0.97 (95% CI 0.95–0.99) and differentiate active PTB from LTBI with AUC 0.94 (95% CI 0.92–0.96) (Figure 5A). The scores of TB patients decreased significantly after effective treatment (Figure 5B).

The transcription of the four genes decreased gradually with effective TB treatment in GSE40553 and GSE56153 databases (Supplementary Image 2). Therefore, we tested whether TB score of the four genes can be used to assess the treatment response in databases. For the active TB patients under lengthy treatment, the TB score was significantly decreased after treatment (Figure 5C). In GSE56153, the TB scores of patients returned to normal after treatment between healthy control and recovery (Figure 5D, Wilcoxon *p* > 0.05). The results indicate that the four genes can be biomarkers to monitor treatment efficacy. In summary, the four gene’s signatures are excellent specific TB diagnostic biomarkers in the pilot test. However, multiple center clinical studies with more cases should be conducted in the future.

## Discussion and Conclusion

Novel biomarkers for rapid and reliable TB diagnosis and treatment efficacy monitors are urgently needed to reduce or eliminate the global burden of TB. Here, we used both a prospective study and public datasets with more than 1,000 whole blood patient samples across a range of ages and countries to find diagnostic biomarker genes for the diagnosis of active TB. We found a four-gene set (UBE2L6, BATF2, SERPING1, and VAMP5), and cross-validated it in five additional independent whole blood datasets. The results showed that the four-gene set is robust for the diagnosis of active PTB with other pulmonary TB and HC, while the diagnostic performance is not affected by HIV status based on the datasets. In addition, the four-gene set can help to distinguish active TB from LTBI, which is usually accomplished by TST or IGRAs test. More importantly, we have confirmed that the accuracy of the novel detection method is significantly higher than that of the IGRAs test. The transcription levels of the four-gene decreased stepwise upon effective treatment and could also be biomarkers to monitor treatment efficacy. Whether they can be biomarkers for treatment failure or relapse remains to be determined. Our data indicated that the combination of the four gene set can reach sensitivity of 88 and 78% specificity for the PTB which were significantly higher than 75.3% of sensitivity and 69.1% of specificity by T-SPOT.TB in the same cohort population. The novel biomarkers can reach as high as 100% sensitivity by parallel of BATF2 and VAMP5 and 89.5% specificity by combination of SERPIG1, UBE2L6, and VAMP5. The most effective three gene combination is BATF2-SERPIG1-VAMP5 with 77% specificity and 91% sensitivity for the diagnosis of PTB. The most effective combination of two genes is SERPIG1-VAMP5 (76% specificity and 86% sensitivity).

The host immune response is crucial for the outcome of active TB. However, the genes and pathways involved in host immune response to *M. tuberculosis* infection or persistence remain elusive. Based on Genecards annotation, the four genes are all involved in well- established immune response, but very few studies associated them with TB. The protein-protein interaction network of the four genes constructed *via* STRING-DB database showed that they are strongly associated with ubiquitination, immune cell differentiation, complement activation, and vesicle trafficking (Supplementary Image 3), which are important cellular responses during host interaction with *M. tuberculosis*. BATF2, also called SARI, is a member of the BATF subfamily of basic leucine zipper proteins regulated by interferon and an inhibitor of AP-1 in human cells (Haiqing et al., 2011), which controls the differentiation of lineage-specific cells in the immune system (Murphy et al., 2013), and Batf2/Irf1 induces inflammatory responses in mycobacterial infection (Sugata et al., 2015). Ubiquitin conjugating enzyme E2 L6 (UBE2L6) serves as an E2 enzyme for post-translational addition of an ubiquitin-like protein ISG15 which is vital for antiviral immunity (Skaug and Chen, 2010) and is involved in the type-I interferon response in active TB disease (Ottenhoff et al., 2012). Vesicle-associated membrane protein 5 (VAMP5) is a member of the SNARE protein family, which regulates the docking and fusion of intracellular membrane vesicles (Hong, 2005) and is involved in the development or function of the respiratory system (Ikezawa et al., 2018). VAMP5 controls intracellular transport events, including endocytosis, exocytosis, and internal recycling (Tajika et al., 2014). SERPING1-encoded serpin peptidase Inhibitor (C1Inh), a member of a large family of serine proteases, can influence the complement C1q levels which can mark active disease in human tuberculosis (Cai et al., 2014; Horwitz et al., 2019). By single cell RNA-seq transcriptome of patients with tuberculosis, we found that four genes are highly expressed in white blood cells of patients with tuberculosis. In general, the levels of these four genes in CD14^+^ or CD16^+^ monocytes show the highest trend, among which VAMP5 is relatively higher. This is consistent with the role of monocytes in tuberculosis bacteria. VAMP5 is involved in vesicle transport and has the highest level in monocytes. In addition, the complement activation pathway may also be involved in the elimination of tuberculosis. The sequence-structure-function of the found protein is closely related to its predicted role in tuberculosis. The specific high expression of these genes in TB patients may suggest that they play an important role in the immune response against tuberculosis. Our ongoing study found that the inhibition of BATF2 can benefit the host, suggesting a promising drug target. In addition to the four genes, 63 other key genes we identified were intensively associated with immune response by functional enrichment analysis. Further exploring the immune roles of the 63 genes is worthwhile and might provide more biomarker candidates.

The datasets used in our study have been used by other teams to explore TB diagnostic biomarkers. There is surprisingly little overlap between our results and other reports. Kaforou and colleagues (Myrsini et al., 2013) identified a 44-transcript signature which can distinguish PTB from other diseases (including only one of our genes, SERPING1) and a 27-transcript signature which can distinguish TB from latent TB (including only one of our genes, VAMP5). Berry et al. (2010) found an 86-gene signature which is related to neutrophil-driven type I interferon (no overlap with our four genes) and can discriminate PTB from other inflammatory and infectious diseases. Bloom et al. (2013) identified 144-transcript signature which distinguished PTB from other lung diseases and controls (none of our four genes in it). Anderson et al. (2014) assessed transcript signatures in children and found a 51-transcript signature for distinguishing TB from other diseases (including only one of our genes, VAMP5) and 42-transcript signature for distinguishing TB from latent TB infection (none of our four genes were in it). Bloom et al. (2012) reported an active TB 664-transcript signature and a treatment-specific 320-transcript signature significantly diminished after 2 weeks of treatment. Zak et al. (2016) identified a 16 gene signature which can predict tuberculosis progression. The size of their gene panel is too large to be clinically affordable or actionable for rapid qRT-PCR-based assay. In contrast, our four genes can differentiate active TB from latent TB and other diseases. The four-gene set will reduce the cost in its clinical qRT-PCR-based diagnosis. Similarly, Costa et al. (2015) found a three-gene set (GZMA, GBP5, and FCGR1A), Sutherland and colleagues (Maertzdorf et al., 2016) found a four-gene set (GBP1, IFITM3, P2RY14, and ID3), Ottenhoff et al. (2012) found a three-gene set (IL15RA, UBE2L6, and GBP4), Sweeney et al. (2016) found a three-gene set (GBP5, DUSP3, and KLF2), and Roe et al. (2019) found a three-gene set (BATF2, GBP5, and SCARF1) in blood samples that can distinguish TB. But our biomarker genes are different and validated in a Chinese population.

The discrepancy between our result and other reports might have resulted from the ethnicity or the bioinformatic pipelines. Our approach uniquely integrated three bioinformatics methods and validated the results by prospective study in a Chinese population. We explored transcript signatures *via* integrating differential expression genes, co-expression networks, and expression trends, which can interpret the expression data from multiple dimensions. This rigorous pipeline might underlie the good performance of the four genes. However, this pipeline might miss some candidate biomarkers. There might be additional biomarker genes which can be included for better performance in regions with low incidence rates of active tuberculosis.

Although there are some reports that clearly affirm that some of these genes can be used as a biomarker for TB diagnosis, the effectiveness of a single gene is flawed. The flexible application of the four genes set that we found is a fast and effective diagnostic method for active TB disease. Moreover, this four genes set can also be used as detection molecules for the treatment effect of TB, and are expected to play an important role in quickly distinguishing PTB from LTBI.

In summary, we demonstrated that the four-gene set (BATF2, UBE2L6, VAMP5, and SERPING1) is a robust blood-based diagnostic for active TB across seven datasets containing more than 1,200 clinical samples, the sensitivity or specificity of which can reach 100%, though the mean AUC = 0.86, sensitivity = 86%, and specificity = 81%. They span a variety of age, infection or exposure status, ethnicity (Sutherland et al., 2014) and genetic backgrounds, and diverse circulating lineages of *M. tuberculosis*. This was further validated in 126 human blood specimens from a Chinese population. The four-gene set can serve as biomarkers to improve clinical diagnosis and treatment response monitoring of TB.

## Data Availability Statement

The datasets presented in this study can be found in online repositories. The names of the repository/repositories and accession number(s) can be found in the article/ Supplementary Material.

## Ethics Statement

The studies involving human participants were reviewed and approved by the study protocols were approved by the Institutional Review Board at the Hospital (K17-022). All participants provided written informed consent prior to participation in the study. The patients/participants provided their written informed consent to participate in this study.

## Author Contributions

YG, XK, ZG, JN, and RZ performed the experiments. YG, LF, and JX analyzed the data. BS and LF diagnosed the patients and collected samples for all clinically related ethical approval. ZG, YG, LF, and JX designed the study and wrote the manuscript. All authors have read and approved the manuscript.

## Conflict of Interest

The authors declare that the research was conducted in the absence of any commercial or financial relationships that could be construed as a potential conflict of interest.

## Supplementary Material

Supplementary Table 1 The 63 genes were found in the DEGs, WGCNA black module, and STEM profiles.

Supplementary Table 2 The transcription of the four genes in PTB, HC, TUMOR, and INFLA.

Supplementary Image 1 Different combinations of four genes showed different advantages in the diagnosis of active TB.

Supplementary Image 2 The transcription of the four genes decreased gradually with effective TB treatment in GSE40553, GSE56153 databases.

Supplementary Image 3 The protein-protein interaction network of the four genes constructed *via* STRING-DB database.

## Abbreviations

BATF2: Basic leucine zipper transcriptional factor ATF-like
GS: Gene significance
MTB: Mycobacterium tuberculosis
MM: Module membership
NIH GEO: National Institutes of Health Gene Expression Omnibus
POCT: Point-of-care tests
qRT-PCR: quantitative real-time PCR
TB: Tuberculosis
IGRAs: interferon γ release assays
STEM: Short Time-series Expression Miner
TST: Tuberculin skin testing.

## References

[B1] Akkaya O. Kurtoglu M. G. (2019). Comparison of conventional and molecular methods used for diagnosis of mycobacterium tuberculosis in clinical samples. *Clin. Lab.* 65. 10.7754/Clin.Lab.2019.190145 31625374

[B2] Anderson S. T. Myrsini K. Brent A. J. Wright V. J. Banwell C. M. George C. (2014). Diagnosis of childhood tuberculosis and host RNA expression in Africa. *N. Engl. J. Med.* 370 1712–1723. 24785206 10.1056/NEJMoa1303657 PMC4069985

[B3] Berry M. P. Graham C. M. McNab F. W. Xu Z. Bloch S. A. Oni T. (2010). An interferon-inducible neutrophil-driven blood transcriptional signature in human tuberculosis. *Nature* 466 973–977. 10.1038/nature09247 20725040 PMC3492754

[B4] Bloom C. I. Graham C. M. Berry M. P. R. Rozakeas F. Redford P. S. Wang Y. (2013). Transcriptional blood signatures distinguish pulmonary tuberculosis, pulmonary sarcoidosis, pneumonias and lung cancers. *PLoS One* 8:e70630. 10.1371/journal.pone.0070630 23940611 PMC3734176

[B5] Bloom C. I. Graham C. M. Berry M. P. R. Wilkinson K. A. Tolu O. Fotini R. (2012). Detectable changes in the blood transcriptome are present after two weeks of antituberculosis therapy. *PLoS One* 7:e46191. 10.1371/journal.pone.0046191 23056259 PMC3462772

[B6] Cai Y. Yang Q. Tang Y. Zhang M. Liu H. Zhang G. (2014). Increased complement C1q level marks active disease in human tuberculosis. *PLoS One* 9:e92340. 10.1371/journal.pone.0092340 24647646 PMC3960215

[B7] Costa L. L. D. Delcroix M. Costa E. R. D. Prestes I. V. Milano M. Francis S. S. (2015). A real-time PCR signature to discriminate between tuberculosis and other pulmonary diseases. *Tuberculosis* 95 421–425. 10.1016/j.tube.2015.04.008 26025597 PMC4475479

[B8] Denkinger C. M. Kik S. V. Cirillo D. M. Casenghi M. Shinnick T. Weyer K. (2015). Defining the needs for next generation assays for tuberculosis. *J. Infect. Dis.* 211(Suppl. 2):S29. 10.1093/infdis/jiu821 PMC4447829 25765104

[B9] Haiqing M. Xiaoting L. Yibing C. Ke P. Jiancong S. Hui W. (2011). Decreased expression of BATF2 is associated with a poor prognosis in hepatocellular carcinoma. *Int. J. Cancer* 128 771–777. 10.1002/ijc.25407 20473897

[B10] Hong W. (2005). SNAREs and traffic. *Biochim. Biophys. Acta* 1744 120–144. 15893389 10.1016/j.bbamcr.2005.03.014

[B11] Horwitz J. K. Chun N. H. Heeger P. S. (2019). Complement and transplantation: from new mechanisms to potential biomarkers and novel treatment strategies. *Clin. Lab. Med.* 39 31–43. 10.1016/j.cll.2018.10.004 30709507 PMC6361534

[B12] Ikezawa M. Tajika Y. Ueno H. Murakami T. Inoue N. Yorifuji H. (2018). Loss of VAMP5 in mice results in duplication of the ureter and insufficient expansion of the lung. *Dev. Dyn.* 247 754–762. 10.1002/dvdy.24618 29330887

[B13] Jiang J. Yang J. Shi Y. Jin Y. Tang S. Zhang N. (2020). Head-to-head comparison of the diagnostic accuracy of Xpert MTB/RIF and Xpert MTB/RIF Ultra for tuberculosis: a meta-analysis. *Infect. Dis. (Lond.)* 52 763–775. 10.1080/23744235.2020.1788222 32619114

[B14] Maertzdorf J. McEwen G. Weiner J. III Tian S. Lader E. Schriek U. (2016). Concise gene signature for point-of-care classification of tuberculosis. *EMBO Mol. Med.* 8 86–95. 10.15252/emmm.201505790 26682570 PMC4734838

[B15] Maertzdorf J. Weiner J. Mollenkopf H.-J. Network T. Bauer T. Prasse A. (2012). Common patterns and disease-related signatures in tuberculosis and sarcoidosis. *Proc. Natl. Acad. Sci.U.S.A.* 109 7853–7858. 10.1073/pnas.1121072109 22547807 PMC3356621

[B16] Marc T. Binita D. Susan D. Nicole R. Benjamin F. Kattia C. B. (2015). Mycobacteria-specific cytokine responses detect tuberculosis infection and distinguish latent from active tuberculosis. *Am. J. Respir. Crit. Care Med.* 192 485–499. 10.1164/rccm.201501-0059oc 26030187

[B17] Murphy T. L. Tussiwand R. Murphy K. M. (2013). Specificity through cooperation: BATF-IRF interactions control immune-regulatory networks. *Nat. Rev. Immunol.* 13 499–509. 10.1038/nri3470 23787991

[B18] Myrsini K. Wright V. J. Tolu O. Neil F. Anderson S. T. Nonzwakazi B. (2013). Detection of tuberculosis in HIV-infected and -uninfected African adults using whole blood RNA expression signatures: a case-control study. *PLoS Med.* 10:e1001538. 10.1371/journal.pmed.1001538 24167453 PMC3805485

[B19] Ottenhoff T. H. Dass R. H. Yang N. Zhang M. M. Wong H. E. Sahiratmadja E. (2012). Genome-wide expression profiling identifies type 1 interferon response pathways in active tuberculosis. *PLoS One* 7:e45839. 10.1371/journal.pone.0045839 23029268 PMC3448682

[B20] Pai M. Schito M. (2015). Tuberculosis diagnostics in 2015: landscape, priorities, needs, and prospects. *J. Infect. Dis.* 211 S21–S28. 25765103 10.1093/infdis/jiu803 PMC4366576

[B21] Qureshi S. Sohaila A. Hannan S. Amir Sheikh M. D. Qamar F. N. (2019). Comparison of Xpert MTB/RIF with AFB smear and AFB culture in suspected cases of paediatric tuberculosis in a tertiary care hospital, Karachi. *J. Pak. Med. Assoc.* 69 1273–1278. 31511711

[B22] Rangaka M. X. Wilkinson K. A. Glynn J. R. Ling D. Menzies D. Mwansa-Kambafwile J. (2012). Predictive value of interferon-γ release assays for incident active tuberculosis: a systematic review and meta-analysis. *Lancet Infect. Dis.* 12 45–55. 10.1016/s1473-3099(11)70210-9 21846592 PMC3568693

[B23] Roe J. Venturini C. Gupta R. Gurry C. Noursadeghi M. (2019). Blood transcriptomic stratification of short-term risk in contacts of tuberculosis. *Clin. Infect. Dis.* 70 731–737. 10.1093/cid/ciz252 PMC7617764 30919880

[B24] Skaug B. Chen Z. J. (2010). Emerging role of ISG15 in antiviral immunity. *Cell* 143 187–190. 10.1016/j.cell.2010.09.033 20946978 PMC2981609

[B25] Steingart K. R. Sohn H. Schiller I. Kloda L. A. Boehme C. C. Pai M. (2013). Xpert^®^; MTB/RIF assay for pulmonary tuberculosis and rifampicin resistance in adults. *Cochrane Database Syst. Rev.* 1:CD009593. 10.1002/14651858.CD009593.pub2 PMC4470352 23440842

[B26] Sugata R. Reto G. Parihar S. P. Sebastian S. Bogumil K. Hajime N. (2015). Batf2/Irf1 induces inflammatory responses in classically activated macrophages, lipopolysaccharides, and mycobacterial infection. *J. Immunol.* 194 6035–6044. 10.4049/jimmunol.1402521 25957166

[B27] Suliman S. Thompson E. Sutherland J. Weiner J. III Ota M. O. C. Shankar S. (2018). Four-gene pan-african blood signature predicts progression to tuberculosis. *Am. J. Respir. Crit. Med.* 197 1198–1208. 10.1164/rccm.201711-2340OC 29624071 PMC6019933

[B28] Sutherland J. S. Loxton A. G. Haks M. C. Kassa D. Ambrose L. Lee J. S. (2014). Differential gene expression of activating Fcγ receptor classifies active tuberculosis regardless of human immunodeficiency virus status or ethnicity. *Clin. Microbiol. Infect.* 20 O230–O238. 24205913 10.1111/1469-0691.12383

[B29] Sweeney T. E. Braviak L. Tato C. M. Khatri P. (2016). Genome-wide expression for diagnosis of pulmonary tuberculosis: a multicohort analysis. *Lancet Respir. Med.* 4 213–224. 10.1016/s2213-2600(16)00048-5 26907218 PMC4838193

[B30] Sweeney T. E. Khatri P. (2016). Blood transcriptional signatures for tuberculosis diagnosis: a glass half-empty perspective–Authors’ reply. *Lancet Respir. Med* 4:e29. 10.1016/S2213-2600(16)30039-X 27304800

[B31] Szklarczyk D. Morris J. H. Cook H. Kuhn M. Wyder S. Simonovic M. (2017). The STRING database in 2017: quality-controlled protein–protein association networks, made broadly accessible. *Nucleic Acids Res.* 45 D362–D368. 27924014 10.1093/nar/gkw937 PMC5210637

[B32] Tajika Y. Takahashi M. Khairani A. F. Ueno H. Murakami T. Yorifuji H. (2014). Vesicular transport system in myotubes: ultrastructural study and signposting with vesicle-associated membrane proteins. *Histochem. Cell biol.* 141 441–454. 10.1007/s00418-013-1164-z 24263617

[B33] Venkatraman E. S. (2015). A permutation test to compare receiver operating characteristic curves. *Biometrics* 56 1134–1138. 10.1111/j.0006-341x.2000.01134.x 11129471

[B34] Wallis R. S. Pai M. Menzies D. Doherty T. M. Walzl G. Perkins M. D. (2010). Biomarkers and diagnostics for tuberculosis: progress, needs, and translation into practice. *Lancet* 375 1920–1937. 10.1016/s0140-6736(10)60359-5 20488517

[B35] Walter N. D. Reves R. Davis J. L. (2016). Blood transcriptional signatures for tuberculosis diagnosis: a glass half-empty perspective. *Lancet Respir. Med.* 4:e28. 10.1016/S2213-2600(16)30038-8 27304799

[B36] WHO (2019). *Global Tuberculosis Report 2019.* Geneva: World Health Organisation.

[B37] Wu X. Tan G. Gao R. Yao L. Bi D. Guo Y. (2019). Assessment of the Xpert MTB/RIF Ultra assay on rapid diagnosis of extrapulmonary tuberculosis. *Int. J. Infect. Dis.* 81 91–96. 10.1016/j.ijid.2019.01.050 30738907

[B38] Zak D. E. Penn-Nicholson A. Scriba T. J. Thompson E. Suliman S. Amon L. M. (2016). A blood RNA signature for tuberculosis disease risk: a prospective cohort study. *Lancet* 387 2312–2322. 10.1016/s0140-6736(15)01316-1 27017310 PMC5392204

