# Supplementary figures and images for: The Evaluation and Validation of Blood-Derived Novel Biomarkers for Precise and Rapid Diagnosis of Tuberculosis in Areas With High-TB Burden
Source: Front Microbiol. 2021 Jun 14;12:650567. doi: 10.3389/fmicb.2021.650567 (PMC8236956; doi:10.3389/fmicb.2021.650567)

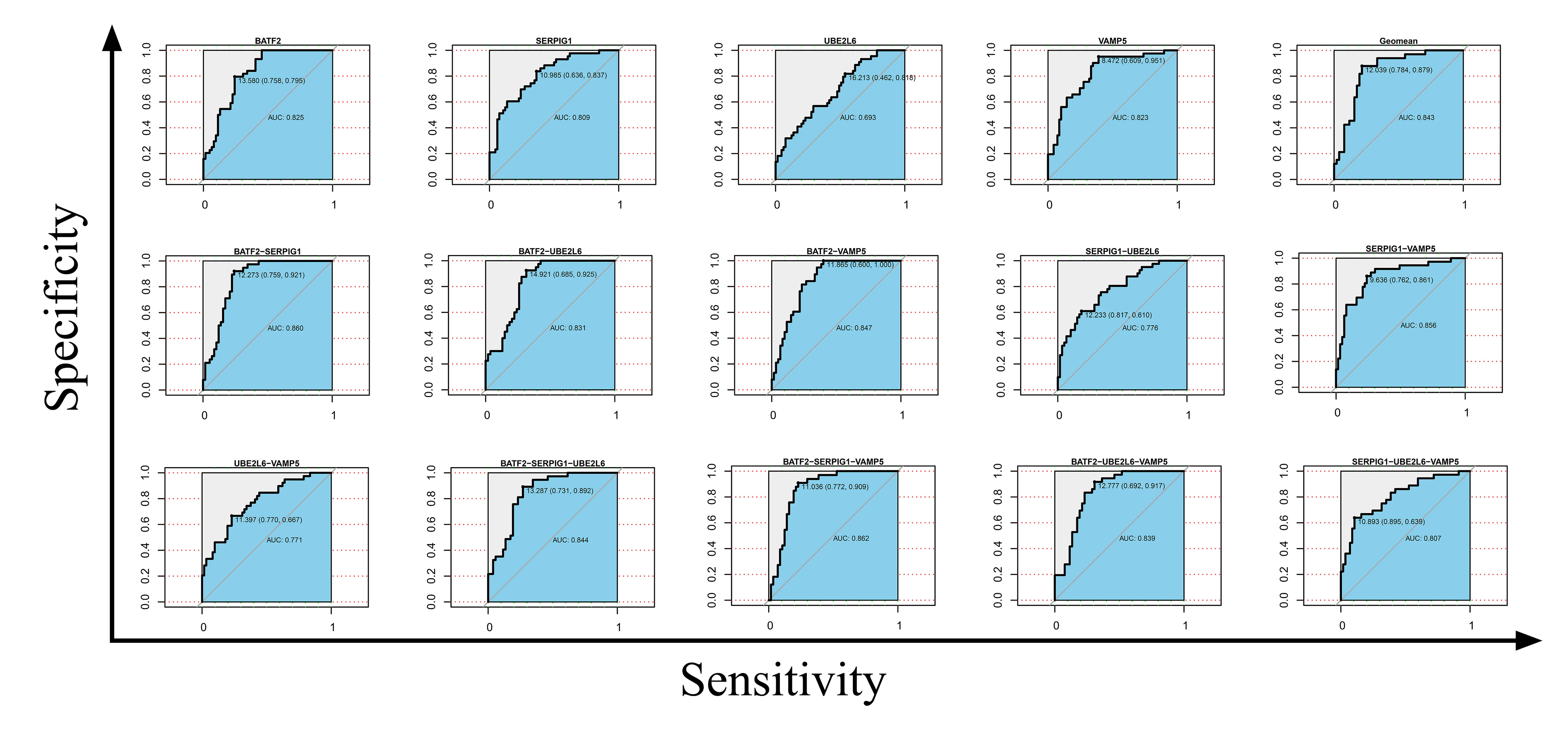

Supplement: Supplementary Image 1 — Different combinations of four genes showed different advantages in the diagnosis of active TB. [file Image_1.TIF]

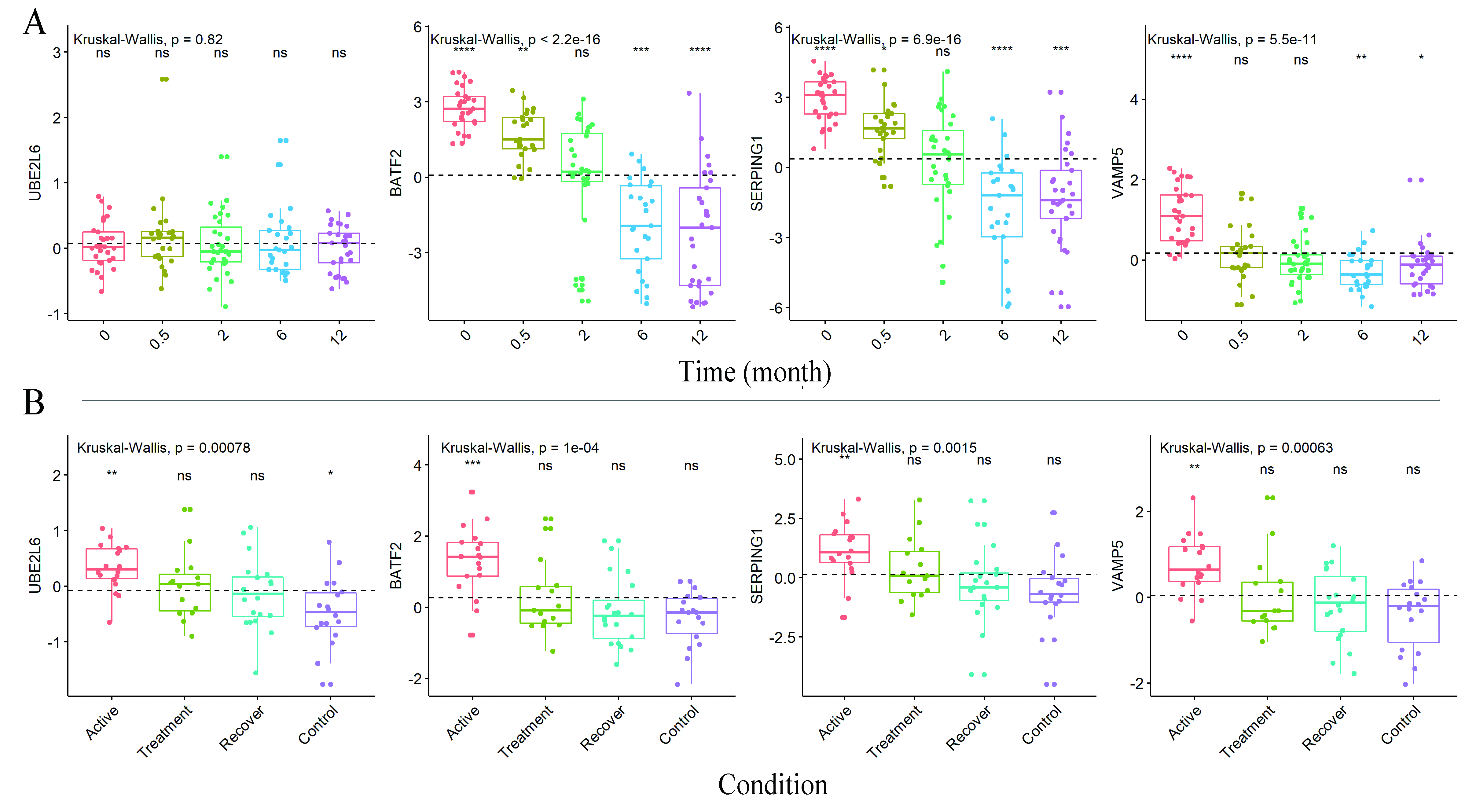

Supplement: Supplementary Image 2 — The transcription of the four genes decreased gradually with effective TB treatment in GSE40553, GSE56153 databases. [file Image_2.TIF]

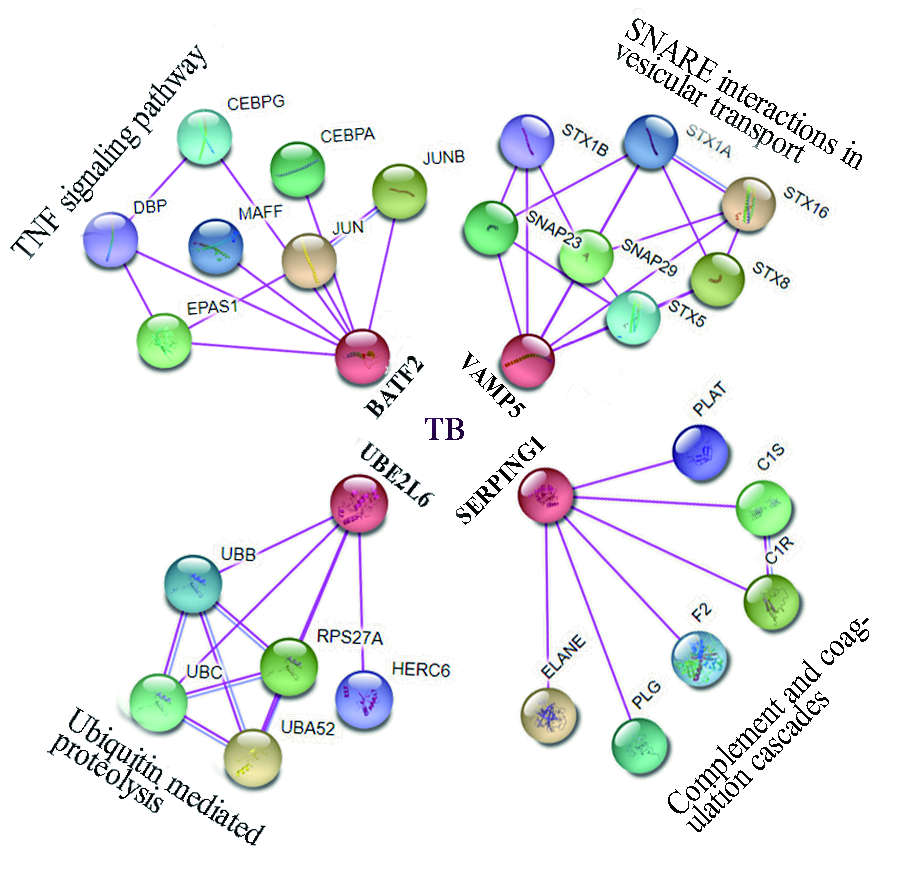

Supplement: Supplementary Image 3 — The protein-protein interaction network of the four genes constructed via STRING-DB database. [file Image_3.TIF]
